# Changes in Purpose in Life and Low-Grade Chronic Inflammation Across Older Adulthood

**DOI:** 10.1177/00914150231196098

**Published:** 2023-08-29

**Authors:** Irene Giannis, Carsten Wrosch, Heather Herriot, Jean-Philippe Gouin

**Affiliations:** 1 5618 Concordia University, Montreal, Canada

**Keywords:** purpose in life, low-grade chronic inflammation, aging, early and advanced old age

## Abstract

**Background:** Older adults often experience an increase in low-grade chronic inflammation. Purpose in life could act as a protective factor as it is associated with beneficial health outcomes. Purpose in life may exert part of its adaptive function by promoting persistence in goal pursuit. During older adulthood, however, when many individuals experience an increase in intractable stressors and declining resources, the adaptive function of purpose could become reduced. **Purpose:** We examined whether the association between inter- and intra-individual differences in purpose in life and chronic inflammation differed across older adulthood. **Method:** We assessed four waves of data among 129 older adults (63–91 years old) across 6 years. **Results:** Hierarchical linear modeling demonstrated that within-person increases in purpose in life predicted reduced levels of chronic inflammation in early old age (25th percentile or 73 years, *coefficient* = −.016, *p < *.01), but not in advanced old age (75th percentile or 81 years, *coefficient* = .002, *p = .*67). Between-person differences in purpose were not related to chronic inflammation. **Conclusions:** These results suggest that greater within-person increases in purpose may protect health processes particularly in early old age but become less effective in advanced old age.

## Introduction

The aging population represents the fastest growing segment of the human population (United Nations, 2017). Older adults’ emotional, social, and physical functioning can remain protected, with such functioning especially maintained in early old age (Baltes & Smith, 2003). However, many older adults experience an increase in age-related chronic stressors, including the onset of physical illness, functional disability, or irrevocable social losses (Heckhausen et al., 2019; Scheibe & Carstensen, 2010). Advanced old age, in particular, represents a life period in which people confront limits to their functional capacity and increasing levels of intractable stressors (Baltes & Smith, 2003; Gerstorf et al., 2011; Sutin et al., 2013).

The emergence of various age-related stressors across older adulthood could contribute to a normative increase in older adults’ physical health problems (for stress and disease, see Cohen et al., 2007). An important physiological process that could enhance older adults’ risk of experiencing stress-related physical health problems relates to their levels of low-grade chronic inflammation (Franceschi & Campisi, 2014). To this end, low-grade inflammation may exert important downstream implications and act as a major pathophysiological mechanism through which stress affects physical health (Miller et al., 2002; Piazza et al., 2018). Older adults may be particularly vulnerable to the effects of low-grade chronic inflammation as research has demonstrated age-related functional changes to the immune system (Aw et al., 2007). These physiological changes in turn, are associated with increased levels of low-grade chronic inflammation in older adulthood and increased risk of inflammation-related disease (Aw et al., 2007). In support of this possibility, low-grade chronic inflammation has been associated with age-related disease including the onset of neurodegenerative disorders, diabetes, heart disease, arthritis, and certain cancers (Allin & Nordestgaard, 2011; Danesh et al., 2004; Franceschi & Campisi, 2014; Willerson & Ridker, 2004).

Given the adverse health effects of low-grade chronic inflammation, the identification of psychological factors that may be protective seems crucial to enhance quality of life in older adulthood. One psychological factor that may protect older adults’ health-related functioning in the context of stressors is purpose in life. High levels of purpose entail a sense of direction and meaning in a person's life and the extent to which a person engages in meaningful and valued goals and activities (Ryff, 1989; Ryff & Keyes, 1995; Scheier et al., 2006). Purpose in life has been associated with improved well-being and physical health outcomes across the lifespan including reduced risk of depression, increased longevity, lower levels of myocardial infraction, stroke, and all-cause mortality (e.g., Hill & Turiano, 2014; Kim et al., 2013a, 2013b; Kim et al., 2019a; Mezick et al., 2010). In addition, studies have documented a direct association between purpose in life and chronic inflammation. For example, greater purpose in life has been related with lower circulating levels of interleukin-6 (IL-6) and fewer soluble IL-6 receptors in samples of younger and older adults (Friedman et al., 2007; Morozink et al., 2010; Rohleder, 2014; Ryff et al., 2004). However, this research has been limited to cross-sectional studies, and to date, there has only been one longitudinal study showing a protective effect of purpose in life on chronic inflammation over time (Guimond et al., 2022).

Purpose in life may protect physical health through different pathways. For instance, individuals with greater purpose may engage in more health-supportive behaviors, including more frequent attendance to medical check-ups and physical examinations as well as greater engagement in moderate to vigorous physical exercise (Hooker & Masters, 2016; Kim et al., 2014). Purpose may also directly facilitate health-relevant psychological processes associated with psychological well-being. In this regard, people who are engaged in valuable goals whose pursuit they enjoy may experience high levels of emotional well-being and life satisfaction. Supporting this assumption, a large body of research has linked purpose in life with increased levels of positive emotions and life satisfaction (e.g., Mascaro & Rosen, 2005; Ryff & Keyes, 1995; Scheier et al., 2006). Furthermore, purpose in life has been associated with decreased levels of negative emotions and lower levels of perceived stress. Such associations may occur if people with higher levels of purpose in life perceive stressors as less difficult or are less reactive to stressors (Hill et al., 2018; Kim et al., 2019b).

Purpose in life may further protect physical health by triggering effective behavioral responses in the self-regulation of personal goals. As posited by expectancy-value models of motivation, engagement and persistence in goal pursuits should depend on the extent to which a person values an activity (Atkinson, 1964; Feather, 1982). From this perspective, valued goals promote goal engagement and persistence, even when significant obstacles are encountered (Scheier et al., 2006). Since people with high purpose in life pursue personally valuable goals, they can be expected to engage in approach-oriented and problem-solving behaviors to attain important goals or counteract emerging problems and stressors. Consistent with this assumption, purpose in life has been associated with greater active and problem-solving behaviors among older adults who experienced severe and pressing stressors such as knee-replacement surgery or caregiving stress. In turn, these active coping strategies predicted long-term physical health outcomes (Kling et al., 1997b; Smith & Zautra, 2000).

Of importance, the emotional and behavioral concomitants of purpose in life could have important downstream implication for physical health by modulating older adults’ risk of exhibiting patterns of chronic inflammation (Guimond et al., 2022). In this regard, it is reasonable to assume that purpose in life may influence levels of chronic inflammation over time through the various affective and behavioral pathways described above. That is, the positive emotional consequences of purpose in life including higher levels of positive affect, life satisfaction, and lower levels of perceived stress could buffer the adverse effects of stressful experiences on HPA-axis and immune responses (Cohen & Pressman, 2006; Mascaro & Rosen, 2005; Pinquart, 2002; Rohleder, 2014; Ryff, 1989; Scheier et al., 2006). In a similar vein, active coping tactics could reduce stress reactivity and contribute to lower levels of inflammation (Barlow et al., 2017). In support of these ideas, research has linked purpose with reduced cortisol response and allostatic load, two factors that are thought to regulate immune responses such as low-grade chronic inflammation (Fogelman & Canli, 2015; Heffner, 2011; Kiecolt-Glaser et al., 2003). Reduced endocrine and immune responses are consequently thought to reduce risk of disease and improved psychological and physical well-being across the lifespan.

### Effects of Individual Differences or Within-Person Fluctuations in Purpose in Life?

Although the previous discussion documents an established association between purpose in life and health-related outcomes, it is less known whether such an association is driven by between-person differences or within-person changes in purpose in life. In fact, to date most of the extant literature has examined individual differences in purpose and few studies considered the health effects of within-person changes or fluctuations (e.g., Kling et al., 1997a). Furthermore, most of these studies were cross-sectional, rendering it impossible to examine the unique variance in health-related outcomes explained by within- and between-variability in purpose. Notably, within-person variability in psychological constructs can substantially predict health-relevant outcomes (Voelkle et al., 2014), and explain a significant proportion of variance above and beyond between-person differences (Ong & Ram, 2017). In fact, research has shown that purpose can fluctuate in response to major life transitions and adaptations to a person's environment (Kling et al., 1997a).

While it is likely that both between and within-person differences in purpose explain important variance in health-related outcomes, this assumption cannot be verified without a longitudinal analysis that disentangles both effects. For instance, if an event occurs that causes a marked upward shift in a person's purpose, it can be expected that more positive affect will be experienced as well as greater motivation to engage in problem-solving behavior when confronted with goal-related problems (see also, Atkinson, 1964; Feather, 1982), potentially predicting improved health outcomes. As such, it is possible that the health effects reported in the extant cross-sectional literature were driven by within-person fluctuations in purpose, although these effects may have been interpreted as resulting from between-person differences. This possibility highlights the necessity of longitudinal research to elucidate the variance in health-related outcomes explained by both sources of variation in purpose.

### Purpose in Life Across Older Adulthood

Another limitation of the extant research relates to the paucity of work on age effects of purpose in life on health-relevant outcomes across older adulthood. In this regard, we note that age-comparative research documents a substantial normative decline in purpose in life from midlife to old age (Pinquart, 2002; Ryff & Keyes, 1995; Ryff & Singer, 2008; Springer et al., 2011). The reasons for such a decline are varied and could derive from the onset of retirement to the loss of important social roles (e.g., active parenting; Ko et al., 2016). However, little is known about the trajectory of purpose across old age. In this regard, one meta-analysis suggests an age-related decline in purpose in advanced old age (Pinquart, 2002). Consistently, more recent longitudinal data demonstrate that purpose in life declined across older adulthood over an 8-year study period (Hill & Weston, 2019).

In addition to evidence suggesting an age-related decline in purpose across older adulthood, preliminary research also suggests a weakened association between purpose in life and indicators of quality of life in older adulthood. For instance, a meta-analytic review demonstrated that the association between purpose in life and fewer depressive symptoms was attenuated as people advanced in age (i.e., in participants beyond the age of 70; Pinquart, 2002). Given the important positive association between depressive symptoms and inflammatory processes (Howren et al., 2009), the association between purpose in life and inflammation may also become reduced in advanced old age.

Moreover, there are important theoretical reasons that suggest a diminishing effect of purpose across older adulthood. Motivational Theory of Lifespan Development argues that the adaptive value of self-regulatory strategies depends on opportunities and constraints to overcome stressors (Heckhausen et al., 2010; Heckhausen et al., 2019). Substantial research demonstrates that in adulthood and midlife, engaging in active coping and problem-solving behaviors improves psychological and physical well-being (e.g., Wrosch et al., 2000). However, in older adulthood, and especially in advanced old age, opportunities to address such stressors typically decline, and the frequency of intractable stressors and irreversible losses typically increases (Gerstorf et al., 2011; Heckhausen et al., 2019; Smith et al., 2002). As such, research suggests that the adaptive value of active coping strategies declines across older adulthood (e.g., Wrosch et al., 2000). Considering that one of the main adaptive functions of purpose in life is related to the activation of behavioral adaptation processes that promote persistence and contribute to overcoming stressors (Scheier et al., 2006), the beneficial effects of purpose in life on levels of chronic inflammation could remain paramount in early old age, when stressors often remain manageable. By contrast, the beneficial effects of purpose may become reduced or even absent in advanced old age when individuals face an increasing number of intractable stressors. Instead, people in advanced old age may need to engage in psychological processes that facilitate disengagement from unattainable goals to protect their self-perceptions and emotional resources (Barlow et al., 2017; Heckhausen et al., 2010). In support of this argument, recent research documents that while the protective effects of other self-regulation processes that foster goal pursuit (e.g., optimism) become increasingly reduced in advanced old age (Wrosch et al., 2017), self-protective processes (e.g., self-compassion) become paramount in this age group (Herriot & Wrosch, 2021).

### The Present Study

This longitudinal study examined associations between purpose in life and a marker of low-grade chronic inflammation (i.e., C-reactive protein [CRP]) in an age-heterogeneous sample of community-dwelling older adults. As there is a paucity of research examining the health effects of purpose across older adulthood, we examined the associations between purpose and chronic inflammation in early and advanced old age. Furthermore, to address the lack of research on within-person changes, we investigated the effects of both between- and within-person variability in purpose in life on inflammation. To this end, we examined whether between-and within-individual variability in purpose in life would exert age-differential effects across older adulthood. We hypothesized that within-person increases and between-person levels in purpose in life would predict reduced levels of chronic inflammation in early old age. By contrast, we expected that the protective effect of increased and high levels of purpose in life on chronic inflammation would be significantly reduced or absent in advanced old age.

## Method

### Participants

The present study is based on the longitudinal Montreal Health and Aging Study. Participants were recruited through advertisements in local Montreal newspapers. To be eligible for inclusion in the study, participants had to be at least 60 years old at the time of recruitment. A sample of 215 participants was recruited at baseline. Participants were followed up every 2 years (T2: *n =* 184; T3: *n =* 164; T4: *n =* 136; T5: *n =* 125; T6: *n =* 95; T7: *n =* 86). We considered all participants who participated at T4 through T7 of the study, as we began assessing markers of chronic inflammation only at T4. We excluded participants who did not provide data on at least one score of chronic inflammation **(***n* = 7) or purpose in life (*n* = 2). Participants who provided only one score for purpose in life or inflammation did not differ on any main study variable from participants who provided multiple data points (|*t*s| < 1.14, *p*s > .23). Excluded participants did not differ from included participants on any main study variable used in this study (|*t*s| < 0.50, *p*s > .58). After exclusion of these participants, the final sample consisted of 129 participants. Study attrition over T4–T7 of our study sample was attributable to death (*n =* 21), refusal to participate (*n = *15), illness (*n = *2), unable to contact (*n =* 12) unable to follow directions (*n =* 2), or personal reasons (*n* = 1). Participants who dropped-out of our study sample were significantly older at baseline (*M *= 73.63, *SD *= 6.78) than participants who remained (*M *= 71.65, *SD *= 5.35; *t*[134.43] = 2.23, *p *< .05).

### Materials

Low-Grade Chronic Inflammation. Chronic inflammation was measured by assessing CRP levels from T4 to T7. A single use lancet was used to obtain up to three drops of blood, which were collected on filter paper. The collected samples were allowed to dry and subsequently stored in a freezer. The samples were sent for analysis at the Laboratory for Human Biology Research at Northwestern University, where a high sensitivity enzyme immunoassay was utilized (Rueggeberg et al., 2012). The reliability and sensitivity of CRP as measured by single whole blood drops has been demonstrated to be high, and correlations between this method and CRP as derived from venous blood are high (e.g., *r* > .85; McDade et al., 2004). We excluded one CRP score, which was greater than 10 as it may be indicative of acute infection (Pearson et al., 2003). In total, 121 participants had valid CRP data at T4, (T5: *n *= 101; T6: *n *= 77; T7: *n* = 66). At these time points, CRP was positively skewed, demonstrating non-normality of the variable (Tabachnick & Fidell, 2018). To improve the normality of this variable, CRP was log transformed at each time point. The averaged inter-assay coefficient of variation was 8.24 and the ICC was .58.

Purpose in Life. Purpose in life was collected from T4 through T7 and was assessed with the Life Engagement Test (LET; Scheier et al., 2006). The LET assesses the degree to which individuals engage in meaningful and valuable activities in their daily lives. The scale consists of six items, three positively worded items (e.g., “To me, the things that I do are worthwhile”) and three negatively worded items (e.g., “There is not enough purpose in my life”). After reverse coding the negatively formulated items, sum scores were calculated at each wave, where higher scores indicated greater purpose. Across study waves, internal consistency was adequate (αs = .73 to .80; *ICC* = .69). We averaged the scores of purpose in life across waves to obtain an indicator of interindividual differences in levels of purpose in life across the study period.

Covariates. The covariates in this study were assessed at T4 and included age, sex, body mass index (BMI), chronic illness, socioeconomic status (SES), and medication use. Sex was coded as 0 *(Male),* 1 *(Female).* To calculate BMI, participants reported their weight in pounds, and their height in inches. BMI was subsequently calculated by dividing weight in pounds by height in inches squared and multiplying the term by a constant. Chronic illness was assessed with a measure used in previous research, asking participants to report the presence of 17 different chronic illnesses (e.g., high blood pressure, arthritis, cancer, diabetes, Wrosch, Schulz, et al., 2007) and was calculated by computing the number of chronic illnesses reported. Perceived SES was reported with a visual ladder, where 1 indicated the lowest rank, and 10 indicated the highest. Reported income ranged from 0 *(less than $17,000 CAD)* to 5 *(more than $85,000 CAD).* To control for the possibility that certain medications can influence the inflammatory response, we used pre-coded data from the study baseline measurement point (T1; see Rueggeberg et al., 2012). Participants were coded as 0 (*taking no medications)* or 1 (*taking one or more medication(s)* that can either directly impact levels of chronic inflammation, or indirectly affect such levels through known influences on the hypothalamic–pituitary–adrenal axis (e.g., anti-inflammatory drugs, beta-blockers, anti-depressants, or glucocorticoids).

### Missing Data

The applied multilevel modeling analysis (using HLM 6.0; Raudenbush et al., 2004) is capable of handling missing data at Level 1 and therefore missing data at Level 1 were not replaced. However, HLM does not permit missing data for Level 2 variables. At T4, seven participants had missing data for perceived SES, and five participants had missing data for BMI and income. Level 2 missing data corresponded to less than 5% of the sample and were replaced with the sample mean (Tabachnick & Fidell, 2018).

### Data Analyses

Preliminary analyses were conducted to describe the sample and compute the zero-order correlations among variables. The study's main hypotheses were tested with hierarchical linear models. The Level 1 model predicted variability in CRP across waves as a function of an intercept, person-centered scores of time (in years from T4), person-centered scores of purpose in life, and a residual term. Person-centering of Level-1 predictor variables was defined in the HLM analyses. In this model, the intercept represented average levels of CRP across study waves. The time and purpose slopes, respectively, referred to yearly change in CRP levels and the extent to which deviation from an individual's average level of purpose in life would be associated with changes in CRP levels. The Level 2 model predicted the intercept and slopes obtained in Level 1 by participants’ chronological age, average purpose, and study covariates (age, sex, BMI, chronic illness, SES, and medication use). In a final step, we examined whether age would moderate the effect of both average levels of purpose on levels of CRP. While age was assessed as a continuous variable, significant cross-level interactions were plotted at the 25th and 75th percentiles for age. Prior to the analyses, Level 2 predictors were standardized, and the results are based on models using restricted maximum likelihood estimation and robust standard errors. Significant cross-level interactions were interpreted using simple slope analyses and regions of significance (Preacher et al., 2006). Simple slopes were calculated as a function of low and high values of Level 2 moderator variables: constituting the 25th and 75th percentiles, respectively.

## Results

### Preliminary Analyses

The means, standard deviations and percentages of the main study variables are reported in Table 1. At T4, the first used measurement point, participants were on average 78 years old (age range = 63–91), and slightly more than half of the participants were female. Participants had an average BMI of 26 and reported, on average, three chronic illnesses. Slightly more than half of the sample reported an average income at or below 34,000, and the mean of perceived SES was slightly over the midpoint of the scale. Note that, participants’ demographics, number of chronic conditions, and BMI were broadly consistent with the Canadian population at the time of data collection (Statistics Canada, 2010).

**Table 1.** Means, Standard Deviations and Frequencies of Main Study Variables (*N* = 129).

Constructs	Mean (SD) or percentages
Average purpose in life (T4–T7)	23.13 (3.30)
Average C-reactive protein (T4–T7; log mg/L)	1.51 (1.28)
Age (T4)	77.51 (5.15)
Perceived social status (T4)	6.12 (1.84)
Body mass index (T4)	25.92 (4.18)
Chronic illness (T4)	2.78 (1.87)
Medications (%; T1)	77.5
Female (%; T4)	53.50
Income (%; T4)	
Less than 17,000	12.40
17,000–34,000	37.20
34,000–51,000	31.80
51,000–68,000	5.40
68,000–85,000	8.50
More than 85,000	0.80

*Note.* The sample size for BMI, income, and perceived social status was slightly reduced due to missing data for these constructs.

The zero-order correlation among the main study variables are reported in Table 2. Higher average purpose in life was associated with a younger age, greater perceived SES, lower BMI, and less chronic illness. In addition, higher average CRP levels were associated with greater BMI, and lower perceived SES. Higher BMI was associated with greater chronic illness. Lower perceived SES was associated with less reported income. Finally, identifying as female was associated with a lower BMI and lower reported income.

**Table 2.** Zero-Order Correlations of Main Study Variables (*N* = 129).

	1	2	3	4	5	6	7	8
1. Average purpose in life								
2. Average C-reactive protein	−.07							
3. Age	−.20*	−.06						
4. Sex	−.04	−.04	.12					
5. Perceived SES	.19*	−.23*	−.03	−.07				
6. Income	.17	−.15	−.07	−.23*	.33**			
7. Body mass index (BMI)	−.18*	.18*	−.13	−.19*	−.06	.02		
8. Chronic illness	−.19*	−.03	−.00	−.13	−.12	−.09	.25**	
9. Medications	−.13	−.03	.09	−.18	.03	.04	.20*	.45**

*Note.* The sample size for BMI, income and perceived was slightly reduced due to missing data for these constructs. Higher values indicate being female.

*p ≤ .05; **p ≤ .01.

### Main Analyses

The results of the HLM Level 1 model are reported in Table 3. The CRP intercept of the Level 1 model was significant, suggesting that average levels of CRP across waves were different from zero. The analysis also showed a significant effect for the within-person time slope, indicating that CRP levels linearly increased over time.^1^ No significant effect of the within-person purpose in life slope was obtained in the Level 1 model, suggesting that within-person changes in purpose in life across waves were not significantly associated with changes in chronic inflammation in the entire sample. The Level 1 model further showed significant variation in participants’ CRP intercept values (*χ*^2 ^= 421.24, *p* < .001), indicating reliable individual differences in average levels of CRP across study waves. Purpose in life slope values (*χ*^2 ^= 76.50, *p* = .34), and time slope values (*χ*^2 ^= 89.42, *p* = .08) also showed considerable variation across participants, although the variability did not reach significance.

**Table 3.** Results from Hierarchical Linear Models Predicting CRP as a Function of Within-Person Changes in Purpose in Life, Time, Age, and Covariates (*N* = 129).

C-reactive protein (log mg/L)						
	Intercept (average levels)		Slope (time)		Slope (purpose in life)	
	Coefficient (SE)	T-ratio	Coefficient (SE)	T-ratio	Coefficient (SE)	T-ratio
Level 1 (β0; β1; β2)^a^	.341 (.016)	22.407**	.009 (.004)	2.333*	−.005 (.004)	−1.230
Level 2						
Age	−.008 (.015)	−.547	.002 (.004)	.590	.012 (.004)	2.740*
Average purpose	−.002 (.017)	−.149	.000 (.004)	.228	.006 (.004)	1.516
Female	.003 (.015)	.184	−.005 (.004)	−1.180	.002 (.004)	.441
Perceived SES	−.049 (.015)	−3.244*	.000 (.003)	.116	.000 (.004)	.090
Income	−.019 (.015)	−1.275	.002 (.003)	.648	−.002 (.006)	.442
Body mass index	.036 (.020)	1.853	−.003 (.004)	−.953	−.010 (.005)	−2.056*
Chronic illness	−.034 (.019)	−1.778	−.004 (.005)	−.685	.000 (.005)	.076
Medications	.015 (.020)	.741	.009 (.006)	1.620	−.000 (.007)	−.001
Level 2: Interaction effect						
Average purpose×age	.004 (.015)	.241	.004 (.004)	.909	.002 (.006)	.340

SE = standard error; SES = socioeconomic status.

a The first parameter (e.g., *β*_0_) estimated the intercept, which represents participants’ average levels of levels of CRP. The second parameter (e.g., *β*_1_) estimated the time slope, which represents the within-person associations between years in study from T4 to T7 and CRP. The third parameter (e.g., *β*_2_) estimated the LET slope, which represents the within-person associations between changes in purpose in life and CRP. LET = average purpose in life. The Level 1 model had 128 degrees of freedom, and the Level 2 models had 121 degrees of freedom.

*p ≤ .05; **p ≤ .01.

In the Level 2 model, we predicted variability in participants’ average CRP levels (intercept), within-person associations between purpose in life and CRP (purpose in life slope), and longitudinal changes in CRP (time slope) as a function of age, average levels of purpose, and the covariates. The results indicated that of the covariates, only perceived SES predicted the intercept (i.e., average levels) of CRP. Participants who reported lower SES had higher average levels of CRP across study waves. Neither age nor the other covariates significantly predicted the intercept of CRP or the time slope of CRP (see Table 3). In addition, the Level 2 models demonstrated that of the covariates, BMI predicted the association between within-person changes in purpose in life and CRP. Among participants with a high BMI (*coefficient* = −.012, *SE = *.005, *T-ratio* = −2.137, *p *< .05), but not low BMI (*coefficient* = .0001, *SE = *.005, *T-ratio* = .010, *p = *.99), increased levels in purpose in life from one's average predicted a reduction of CRP.

**Figure 1.**
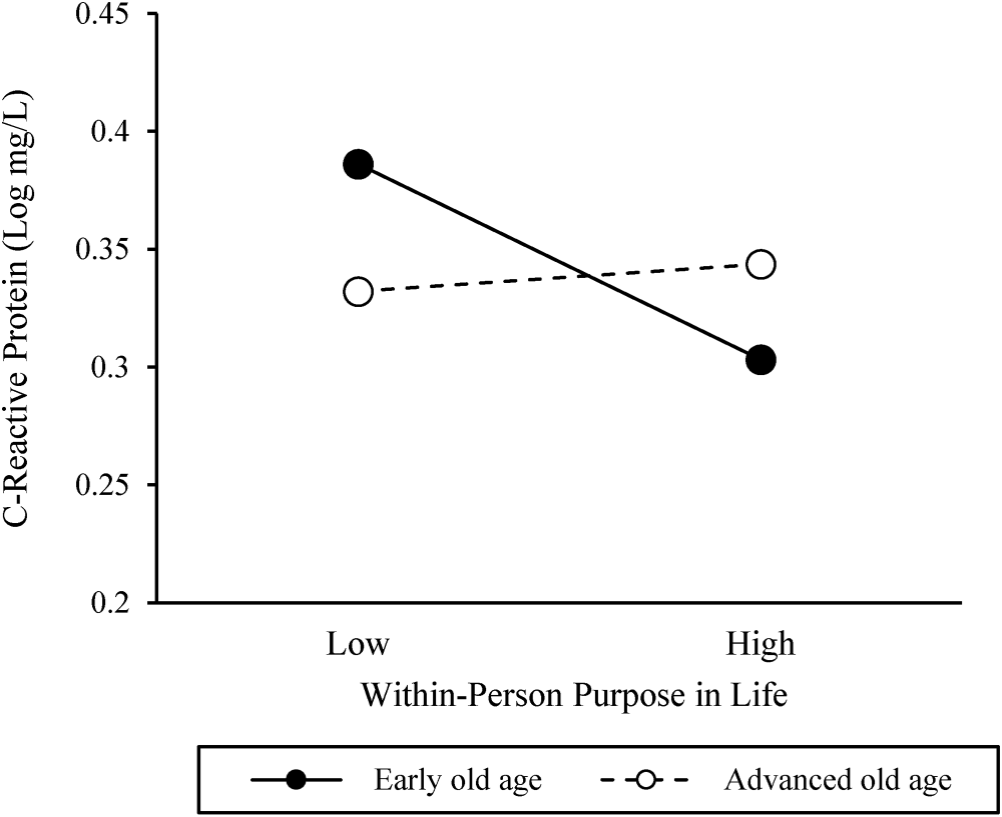
Associations between within-person changes in purpose in life and C-reactive protein among participants in early and advanced old age. The associations were plotted for the 25th and 75th percentiles of the purpose distribution and age distribution (early old age = 73 years; advanced old age = 81 years).

The Level 2 model further showed that, in support of our hypotheses, age moderated the within-person effect of changes in purpose in life on CRP. However, age did not significantly interact with average inter-individual levels of purpose in life in predicting average levels of CRP (see Table 3). The significant cross-level interaction between age and changes in purpose in life in predicting participants’ CRP is illustrated in Figure 1, plotting the association between purpose in life and CRP separately for participants in early and advanced old age, using the 25th (age = 73 years) and 75th (age = 81 years) percentiles of the age distribution as reference points. The shape of the interaction suggests that in early old age, participants had the lowest CRP values in waves they experienced a within-person increase in purpose in life from their average. By contrast, the highest CRP values were observed among participants in early old age in assessments during which they experienced a within-person decline in purpose in life, relative to their average levels. Deviations from one's average level of purpose in life did not appear to be associated with variation in CRP among participants in advanced old age and were located in between the levels of CRP obtained among participants in early old age. Subsequently conducted simples slope analyses supported this interpretation of the data. Within-person increases in purpose in life predicted reduced CRP levels among participants in early old age (*coefficient* = −.016, *SE = *.006, *T-ratio* = −2.796, *p < *.01), but not among their counterparts in advanced old age (*coefficient* = .002, *SE *= .005, *T-ratio* = .416, *p *= .67). Finally, regions of significance were calculated. The results indicated that within-person increases in purpose in life predicted reduced levels of CRP among participants 76 years old and younger (*z* = −.26 *SD* and below the sample mean) and higher levels of CRP among those 89 years and older (*z* =  + 2.30 *SD* and above the sample mean). Including age in the model, above and beyond the covariates, explained an additional 14.3% of the variance in the association between changes in purpose in life and CRP.

## Discussion

The present longitudinal study showed a linear increase in low-grade chronic inflammation over 6 years in a sample of community-dwelling older adults. In addition, a cross-sectional association between purpose in life and chronological age was observed, indicating that individuals in advanced old age experienced lower levels of purpose in life than their counterparts in early old age. Moreover, the analyses demonstrated that within-person increases in purpose, relative to one's average levels, were associated with reduced levels of chronic inflammation in early old age, but not in advanced old age. The obtained moderation effect of age was independent of relevant sociodemographic and health-relevant variables, explaining 14.3% of the variability in the within-person association between purpose in life and chronic inflammation. Neither inter-individual levels of purpose nor the interaction with age predicted average levels or changes in levels of inflammation.

The present study expands on research examining the physiological mechanisms underlying the association between purpose in life and physical health. Numerous studies have reported an inverse association between purpose and inflammatory markers (e.g., Friedman et al., 2007; Friedman & Ryff, 2012; Steptoe & Fancourt, 2019). However, most of this research was cross-sectional, leading to methodological problems including reverse causality (Kim et al., 2019a). To our knowledge, only one study prospectively showed that purpose in life was associated with lower levels of developing CRP among older men over an 8-year study period (Guimond et al., 2022). Thus, the findings of this research suggest that purpose in life may precede, predict, and promote lower levels of inflammation in older adulthood. This longitudinal information is particularly important as older adults are vulnerable to developing inflammatory-related disease (Aw et al., 2007) and purpose in life is a modifiable psychological factor that is amenable to intervention (Van Agteren et al., 2021).

The present findings are also novel in that they address the paucity of research on age effects during older adulthood. To this end, they expand on previous research on age-related changes in purpose in life, demonstrating a cross-sectional decline of purpose from midlife to old age (Pinquart, 2002; Ryff & Keyes, 1995; Ryff & Singer, 2008; Springer et al., 2011). Consistent with this research, our correlational findings document that among older adults, advancing age is associated with lower levels of purpose (see Table 2). As such, it is possible that the age-related decline in purpose may reflect a longer developmental process, one that begins in midlife with substantial changes in employment and loss of important social and family roles (Hill et al., 2015). This process of decline may continue during older adulthood when developmental constraints on goal pursuits increase and individuals have fewer alternative or new goals available to pursue (Heckhausen et al., 2019; Wrosch, Miller, et al., 2007). However, our longitudinal analyses suggest that purpose in life did not significantly decline across time, although there was a trend towards the experience of decreasing purpose in life as older adults advanced in age (see Footnote 1). This discrepancy between cross-sectional and longitudinal analyses could have been observed because our 6-year study period did not match the cross-sectional age distribution and thus did not provide a long enough time interval to capture a significant decrease in purpose in life (for methodological considerations, see also Sliwinski & Buschke, 1999). As such, future longitudinal research over a longer study period may be necessary to replicate the cross-sectional trajectory of purpose across older adulthood.

The reported within-person analysis suggested that increases in purpose in life, relative to one's average, were associated with reduced levels of low-grade chronic inflammation in early old age, but not in advanced old age. These findings are consistent with and corroborate previous literature that associated purpose with lower risk of illness and improved physical well-being (e.g., lower risk of myocardial infraction, stroke, lower circulating levels of Il-6; Friedman et al., 2007; Kim et al., 2013a, 2013b; Kim et al., 2019a, 2019b; Mezick et al., 2010). Furthermore, these findings expand on preliminary research in the field examining the association between purpose in life and indicators of quality of life in older adulthood (Pinquart, 2002). The latter research found that the beneficial effects of purpose on certain outcomes (e.g., depression, Pinquart, 2002) can be reduced as people advanced in age. The present study elaborates on these findings by documenting that increases in purpose in life may also become less effective with advancing age in ameliorating low-grade chronic inflammation, a physiological marker of health that is often associated with depressive symptoms (Howren et al., 2009). It is also important to note the discrepancy in our simple slope and regions of significance findings in advanced old age. While simple slope analyses suggested that the health benefits of purpose in life may become reduced, the regions of significance results suggested a reversal of effect, in which greater within-person purpose may be maladaptive among the oldest individuals (89 years and above) in our sample. While it is possible that emotional turmoil and physiological dysregulation may ensue among older adults who can no longer attain their goals, we note that only two participants in our sample were above the age of 89, rendering the result underpowered (Heckhausen et al., 2019; Wrosch & Scheier, 2020). As such, at present, we cannot assert a reversal of effect of purpose at the oldest ages of older adulthood. Furthermore, it is also possible that the perception of having meaningful goals, even if their attainment becomes increasingly difficult or impossible, could still potentially protect or buffer any negative effects purpose could exert in advanced old age; when engaging in approach-oriented, problem-solving behaviors is no longer helpful in overcoming age-related stressors in the context of unattainable goals (Kling et al., 1997a; Smith & Zautra, 2000; Wrosch et al., 2000, 2003).

We had argued that there could be different pathways through which purpose in life may exert age-differential effects on inflammatory processes during older adulthood. First, older adults who pursue meaningful goals may generally experience greater emotional well-being, life satisfaction and lower levels of perceived stress, which in turn could exert positive downstream implications on health-related physiological processes (Cohen et al., 2007; Hill et al., 2018; Pressman et al., 2019). Later during older adulthood, however, it may become more difficult for some individuals to replace goals that can no longer be pursued with other or new purposeful activities since there may be fewer appropriate or alternative options available (Wrosch, Miller, et al., 2007). As such, since our study showed that participants in advanced old age generally experienced less purpose than their younger counterparts, they may also have benefitted less from the health-relevant emotional consequences of meaningful goal pursuits.

Second, our theoretical rationale suggested that purpose in life may promote health-relevant outcomes by fostering active coping and behavioral responses when people encounter goal-related problems (Kling et al., 1997b). In early old age, when many stressors and goal-related problems can still be potentially addressed, engaging in approach-oriented and problem-solving behaviors may foster overcoming problems and through this process reduce psychological stress, physiological dysregulation, and physical health problems (Wrosch et al., 2002; Wrosch & Schulz, 2008). In advanced old age, however, a normative increase in uncontrollable stressors and reduced resources can limit an individual's opportunities to overcome problems (Baltes & Smith, 2003; Heckhausen et al., 2019). As such, the adaptive value of psychological processes that foster problem-solving behavior, such as purpose in life, may become reduced during the later parts of older adulthood (for corroborative research on other psychological factors, see Wrosch et al., 2017). Regardless of the specific reasons, these findings provide evidence for a lifespan developmental perspective on health, which indicates that the effectiveness of self-regulation factors depends on a person's age-related position in the life course and his or her opportunities and constraints for development (Heckhausen et al., 2010).

It is important to address that our analyses showed age-related effects of older adults’ purpose on inflammatory processes only for within-person associations, but not for between-person associations. In fact, between-person differences in purpose in life were not associated with levels of chronic inflammation, neither in early nor advanced old age. We believe that this pattern of findings emphasizes the importance of studying within-person variation in purpose in life across the lifespan. In support of this conclusion, our analyses showed that ∼30% of the variance in purpose and 40% of the variance in chronic inflammation was located at the within-person level (see ICCs in Method section). In addition, the examination of within-person associations may be important because previous research largely relied largely on cross-sectional data and examined between person-differences in purpose only. Such research, however, cannot determine whether associations between purpose in life and health-related outcomes were observed because of stable between-person differences in purpose or because people recently experienced an increase or decline in purpose in life. As such, it is possible that health effects of purpose in life obtained in previous research were due to within-person increases in purpose, and thus consistent with the findings of our study. Considering that within-person changes in important psychological processes can be as, or more, predictive than stable between individual differences (Voelkle et al., 2014), our findings point to the importance of studying both within and between sources of variability and provide mounting evidence of the importance of within-person variation in psychological constructs (cf. Ong & Ram, 2017).

Since our findings suggest that the health benefits of purpose in life may become less pronounced towards the end of life, it seems important to address how people could effectively manage age-related threats and losses. To this end, we suggest that individuals in advanced old age may need to additionally engage in other psychological processes and coping responses that facilitate disengagement from unattainable goals and protect psychologically against repeated failure experiences (Herriot et al., 2018; Wrosch et al., 2000). This may be particularly important since our data documented a linear increase of chronic inflammation over time, which is consistent with accumulating evidence for substantial declines across different aspects of functioning in advanced old age (Baltes & Smith, 2003; Gerstorf et al., 2011). Supporting this conclusion, previous research has shown that certain psychological factors, such as the capacity to adjust to unattainable goals, avoiding self-blame, being self-compassionate, or positively reframing problematic situations, can protect subjective well-being and physical health among older adults and populations that confront intractable stressors and unattainable goals (Heckhausen et al., 2010; Herriot et al., 2018; Jobin & Wrosch, 2016; Park, 2010; Wrosch & Scheier, 2020).

The study's findings further showed that some of the covariates were meaningfully associated with purpose in life and low-grade chronic inflammation. For example, our cross-sectional results suggested that lower perceived SES predicted higher levels of chronic inflammation. These results are consistent with previous research linking lower SES with increased risk of physiological dysregulation, illness, and mortality through a constellation of environmental and psychosocial factors (Chen & Miller, 2013; Rejineveld, 1998; Seeman et al., 2004). With respect to the relation between SES and inflammation, several mediators including the experience of greater acute and chronic stress, lower levels of positive affect, and poorer health behaviors have been identified as mediating this association (Chen & Miller, 2013; Chiang et al., 2015; Janicki-Deverts et al., 2012). Our cross-sectional results also indicated that a lower perceived SES was associated with less purpose in life. To this end, lower levels of purpose may be experienced by socially disadvantaged individuals if fewer personal and environmental resources needed to pursue meaningful goals are available (Ryff, 1989). Our analyses further suggested that a higher BMI was associated with both low purpose and high levels of chronic inflammation, and the association between purpose in life and chronic inflammation was moderated by older adults’ BMI. The latter effect indicated that among older adults with a high, but not low, BMI, within-person increases in purpose predicted reduced levels of chronic inflammation. Note that a higher BMI has been associated with a greater risk for inflammatory processes, oxidative stress, and associated morbidity (Bondia-Pons et al., 2012; The Global BMI Mortality Collaboration, 2016). However, different from the observed age moderation, there is little evidence that a higher BMI per se could indicate the presence of intractable problems or unattainable goals. As such, it is possible that purposeful goals are especially protective among populations who are at risk of exhibiting chronic inflammation but are still capable of attaining their goals and overcoming goal-related problems.

Finally, the present findings may hold implications for clinicians and practitioners seeking to improve health in older adulthood. Given that enhanced chronic inflammation is associated with several age-related diseases including several types of cancer, metabolic disorders, and neurocognitive diseases (Allin & Nordestgaard, 2011; Danesh et al., 2004; Willerson & Ridker, 2004), our findings suggest that promoting purposeful activities in early old age may be an important contributing factor to physical health. This conclusion underscores the importance for health-care providers to help maintain people's purpose throughout early old age by facilitating the identification of meaningful and valuable goals. In advanced old age, by contrast, such interventions may become less impactful, and clinicians may need to turn to supporting other psychological processes that could make it easier for individuals to adjust to the inability to overcome goal-related problems (e.g., self-protection or goal disengagement, Heckhausen et al., 2019; Wrosch & Scheier, 2020).

## Limitations and Future Research

The present study has different strengths as it is theory-based, assessed longitudinal data and objective indicators of health-related functioning, and provided support for a lifespan perspective on purpose in life and health. Nonetheless, there are also several study limitations that should be addressed in future research. First, although our sample was heterogeneous in terms of socioeconomic background, age, and sex, and broadly consistent with that of the Canadian population (Statistics Canada, 2010), it was relatively small and recruited from a limited geographic region. As such, the obtained findings may not generalize to the population of older adults (e.g., those living in rural areas). We further acknowledge that our study did not collect data related to ethnicity/race and religion, two important factors in multicultural Canadian society, and thus our results may not generalize to the broader population. Future research should replicate the reported findings with larger and more diverse samples.

Second, purpose in life was not experimentally manipulated and causal relations cannot be inferred. Future intervention studies should therefore substantiate our conclusions by experimentally enhancing purpose in life to elucidate causal effects on indicators of low-grade chronic inflammation.

Third, our study did not examine other personality and psychosocial variables that might also protect against low-grade chronic inflammation in older adulthood. For example, optimism and gratitude have been associated with lower levels of inflammation (Carver & Scheier, 2017; Lee & Way, 2019; Liao & Weng, 2018). Furthermore, social factors (e.g., social connectedness or social support) can be associated with physical health (Holt-Lunstad et al., 2010). Importantly, these factors may be related to each other and share variance with purpose in life and inflammation (Liao & Weng, 2018). Future research should therefore incorporate a larger number of psycho-social factors to study their unique contributions to inflammatory processes across older adulthood.

Fourth, the time interval between measurements was about 2 years in our study, which corresponds with our theoretical rational, suggesting that (longer term) declines in purpose and opportunities for goal attainment may underly the observed age-related effects of purpose on inflammatory processes. However, our design was not able to examine whether comparable effects of purpose would occur for shorter time intervals (e.g., day-to-day fluctuations). The latter possibility may also be plausible if short-term changes in purpose influence psychological stress perceptions and should be studied in future research.

Fifth, our study did not examine potential mediators that could explain the longitudinal association between purpose in life and chronic inflammation. Our approach suggests different pathways in which purpose can lead to reduction in CRP, including health behaviors, affective well-being, and reduced stress perception (Hill et al., 2018; Hooker & Masters, 2016; Kim et al., 2014; Pressman et al., 2019**)**. We therefore suggest that future longitudinal research should conduct studies that can examine in fine-grained analyses how emotional and self-regulation processes link changing levels of purpose with health-relevant outcomes across older adulthood.

Sixth, our study examined CRP as a marker of chronic inflammation, and future research should study whether purpose in life extends its age-related effects to other biomarkers of immune and endocrine functioning (e.g., IL-6 or diurnal cortisol patterns).

Finally, the present analysis predicted older adults’ inflammatory responses and did not assess the downstream consequences that chronic inflammation could have on clinical health outcomes. As our theoretical model would predict that purpose facilitates both physiological regulation and physical health, future research should extend the present analyses by examining whether changes in purpose in life are also associated with long-term consequences on physical health outcomes.

## Conclusion

The present study examined the protective effects of changes in purpose in life on preventing low-grade chronic inflammation in a sample of community dwelling older adults. Results showed that chronic inflammation increased throughout older adulthood. Furthermore, individuals in advanced old age reported lower levels of purpose than their relative younger counterparts. In addition, this study showed that within-person increases, but not between-person levels, of purpose in life were associated with reduced levels of chronic inflammation in early old age, but not in advanced old age. These findings support a lifespan perspective on health by pointing to an age-differential role of purpose in life in reducing older adults’ physiological risk for emerging physical health problems. While the experience of purposeful goals may exert beneficial health-related function in early old age, when individuals typically confront goal-related stressors that could be potentially overcome, high levels of purpose in life may be less adaptive in advanced old age, when many older adults confront intractable problems and unattainable goals.

## Notes

1. We note that this increase in CRP was different from purpose in life, which remained relatively stable over the study period, although there was some, but not significant, decline in purpose (*coefficient* = −.072, *SE* *=* .628, *T-ratio* = −1.139, *p* = .26).
